# Change in ^18^F-Fluoromisonidazole PET Is an Early Predictor of the Prognosis in the Patients with Recurrent High-Grade Glioma Receiving Bevacizumab Treatment

**DOI:** 10.1371/journal.pone.0167917

**Published:** 2016-12-09

**Authors:** Shigeru Yamaguchi, Kenji Hirata, Takuya Toyonaga, Kentaro Kobayashi, Yukitomo Ishi, Hiroaki Motegi, Hiroyuki Kobayashi, Tohru Shiga, Nagara Tamaki, Shunsuke Terasaka, Kiyohiro Houkin

**Affiliations:** 1 Department of Neurosurgery, Graduate School of Medicine, Hokkaido University, Sapporo, Hokkaido, Japan; 2 Department of Nuclear-Medicine, Graduate School of Medicine, Hokkaido University, Sapporo, Hokkaido, Japan; Wayne State University, UNITED STATES

## Abstract

**Background:**

Bevacizumab (BEV), a humanized monoclonal antibody, become a currently important chemotherapeutic option for the patients with recurrent glioma. The aim of this retrospective study is to investigate whether ^18^F-Fluoromisonidazole (FMISO) PET have the potential to detect BEV-resistant gliomas in the early-stage.

**Methods:**

We reviewed the FMISO PET and MRI appearances before and 3 to 4 courses after BEV treatment on 18 recurrent glioma patients. FMISO accumulation was assessed by visual inspection and semi-quantitative values which were tumor-to-normal (T/N) ratio and hypoxic volume. MRI responses were evaluated based on RANO (Response Assessment in Neuro-Oncology) criteria. The prognostic analysis was performed in relation to the response assessment by FMISO PET and MRI using overall survival (OS) after BEV application.

**Results:**

After BEV application, MRI revealed partial response in 14 of 18 patients (78%), of which 9 patients also demonstrated decreased FMISO accumulation. These 9 patients (50%) were classified as “MRI-FMISO double responder”. As for the other 5 patients (28%), FMISO accumulation volumes increased or remained stable after BEV treatment although partial responses were achieved on MRI. Therefore, these cases were classified as “MRI-only responder”. The remaining 4 patients (22%) did not show treatment response on FMISO PET or MRI (“non-responder”). MRI-FMISO double responders showed significantly longer OS than that in other groups (median 12.4 vs 5.7 months; *P* < 0.001), whereas there were no overall survival difference between MRI-only responders and non-responders (median OS, 5.7 and 4.8 months; *P* = 0.58). Among the pre-treatment clinical factors, high FMISO T/N ratio was a significant prognostic factor of overall survival in these patients under the assessment of Cox proportional hazard model.

**Conclusions:**

Recurrent gliomas with decreasing FMISO accumulation after short-term BEV application could derive a survival benefit from BEV treatment. Change in FMISO PET appearance can identify BEV-resistant gliomas in early-stage regardless of MRI findings in a comprehensible way.

## Introduction

Although patients with newly diagnosed glioblastoma (GBM) who were treated with bevacizumab did not show increased survival in two recent studies [1,2], bevacizumab (BEV)—a humanized monoclonal antibody that inhibits vascular endothelial growth factor (VEGF)—has become an indispensable chemotherapeutic treatment for patients with recurrent glioma [3,4].

Once BEV is administered, the appearance of tumors on magnetic resonance imaging (MRI) changes dramatically, and the traditional evaluation of treatment response, which is based on the criteria developed by Macdonald *et al* [5], is no longer sufficient. Recurrent high-grade gliomas treated with BEV sometimes simultaneously show regression of contrast enhancement and progression of T2/fluid-attenuated inversion recovery (FLAIR) hyperintensities [6–9]. Thus, the recently published Response Assessment in Neuro-Oncology (RANO) criteria proposes that evaluation of treatment by anti-angiogenic agents should be based on both enhancing T1-weighted MRI sequences and non-enhancing T2-weighted / FLAIR sequences [10]. However, even though response would be assessed by RANO criteria, the relationship among changes in T1-weighted enhancing lesions, non-enhancing FLAIR progression, and overall survival, when patients are treated with anti-angiogenic agents, remains controversial [7,11–13].

Therefore, in addition to the conventional MRI, other objective methods have proven effective in evaluating patient response to BEV treatment [14]. Metabolic imaging using radiolabeled tracers on positron emission tomography (PET) allows for more accurate estimation of the size and extent of the metabolically active tumor. Therefore, it may overcome some of the disadvantages of MRI. Currently, in patients with recurrent high-grade gliomas, two amino acid PET tracers—^18^F-Fluoroethyl-L-tyrosine (FET) [15,16] and 3,4-dihydroxy-6-[^18^F]-fluoro-L-phenylalanine (FDOPA) [17]—have been reported as the promising prognostic metabolic biomarkers in evaluating response to BEV treatment. These studies showed that tumor volume changes, defined as ^18^F-FET or ^18^F-FDOPA, could be strong predictors of the prognosis of patients who receive BEV treatment. However, these amino acid tracers may be insufficient for the early detection of bevacizumab-resistant gliomas, because as a result of the change in tracer uptakes, which were presented as standardized uptake values (SUVs), correlations between “responders” and “non-responders” could not be observed in every study.

In addition to amino acid tracers, hypoxic tracers have become notable PET tracers for evaluating tumor characteristics in several cancers, including gliomas, owing to the fact that hypoxia is a key metabolic factor known to affect treatment outcome. ^18^F-fluoromisonidazole (FMISO) is a representative hypoxia PET tracer [18], and several studies reported the usefulness of FMISO PET specifically in gliomas, concerning differential diagnosis [19], assessment of regional biological aggressiveness [20,21], and prediction of prognosis [22,23]. However, except a single case report [24], no previous investigation has studied the association between FMISO PET findings and BEV treatment for high-grade gliomas.

Several studies have sought to elucidate the mechanisms of various anti-angiogenic agents, one of which induces intratumoral oxygenation through normalization of tumor vasculature [25]. In one study, Valable *et al* showed that ^18^F-FMISO PET in C6 rat glioma models could effectively assess the response of anti-angiogenic treatment with sunitinib [26]. Their study indicated that FMISO can detect changes under the hypoxic condition caused by anti-angiogenic treatment. If it is true, FMISO can potentially become a tool to determine the effect of treatment. However, paradoxically, Hu *et al* recently showed that increasing tumor hypoxia during anti-angiogenic therapy is a novel resistance mechanism, because hypoxia-induced autophagy promotes tumor cell survival [27].

Based on these studies, we reviewed the FMISO PET appearance of tumors in patients with recurrent high-grade glioma who were treated with BEV. We found that, in each case, changes in FMISO accumulation intensity and volume after 3–4 courses of BEV were significant. We found that FMISO measurement of the hypoxic response was a strong predictor of patient prognosis and that the extent of hypoxia before BEV treatment correlated with patient prognosis. These results led us to suggest that FMISO PET may be an effective tool for the early detection of anti-angiogenic therapy-resistant high-grade gliomas. If so, its use may prevent the unnecessary administration of BEV or indicate the necessity for other treatment options at an earlier stage.

## Materials and Methods

### Patients

This retrospective study was approved by the local ethics committee at Hokkaido University Hospital, Sapporo, Japan. Written informed consent was obtained from each patient. We reviewed the medical records of 27 patients with recurrent supratentorial gliomas who were treated with BEV and had accompanying MRI and FMISO PET scans. We excluded patients who underwent surgical resection of recurrent lesions before the administration of BEV. We also excluded patients who had not received conventional radiotherapy and first-line chemotherapy with temozolomide (TMZ) for primary glioma, as well as patients who underwent additional radiotherapy and received BEV simultaneously for recurrent lesions, because FMISO accumulation to the tumor is strongly decreased by radiotherapy, as we reported previously [28].

Eighteen patients met our inclusion criteria. Initial histological diagnosis revealed twelve GBMs, five anaplastic astrocytomas, and one diffuse astrocytoma. Prior to recurrence, all patients had received radiotherapy and TMZ as first-line chemotherapy, according to the Stupp regimen [29]. Before the administration of BEV, all recurrent lesions were clearly detected and measurable by contrast-enhanced MRI.

### Treatment and evaluation of treatment response

Patients were treated with BEV (10mg/kg) every two weeks. Three patients received BEV alone; the remaining 15 patients received BEV as an add-on chemotherapy agent with TMZ. BEV treatment was continued as long as the patient was treated, regardless of treatment response.

To evaluate treatment response, patients underwent MRI and FMISO PET after 3–4 courses of BEV. Thereafter, serial MRI examinations were performed depending on the patient’s condition. We assessed prognostic outcome using overall survival (OS) from the first day of BEV administration.

#### MRI Assessment

To assess the effects of therapy, patients underwent MRI before and during BEV treatment. Standard anatomical MRI sequences included T1-weighted images with and without gadolinium contrast enhancement, T2-weighted images, and FLAIR images. All images were obtained in the axial plane with a 5 mm slice thickness and a 1.5 mm inter-slice distance.

Early radiological responses after 3–4 courses of BEV were evaluated by MRI appearance, based on RANO criteria [10]. Progressive disease was defined as a more than 25% increase in enhancing lesions or a significant increase in T2/FLAIR non-enhancing lesions compared with baseline scan.

In comparison with FMISO appearance of treatment response, the volumes of contrast enhancement on T1-weighted image MRI were calculated by planimetry methods before BEV administration and after 3–4 courses of BEV. The development of T2/FLAIR lesions was evaluated in semi-quantitative fashion as decreased, stable, and increased fashion.

#### ^18^F-FMISO PET protocol and assessment

Patients were not asked to fast prior to FMISO PET scanning. Static PET scanning began 4 hours after 400 MBq of FMISO was intravenously injected, as we reported previously [19]. In this study, we used a Biograph 64 PET-CT scanner (Asahi-Siemens Medical Technologies Ltd., Tokyo, Japan) and a Gemini TF64 TOF-PET/CT scanner (Hitachi Medical Corporation Ltd, Tokyo, Japan). Both scanners were operated in the three-dimensional mode. Computed tomography (CT) scanning for attenuation correction was followed by a 10-minute emission acquisition. Attenuation-corrected radioactivity images were reconstructed using a filtered back-projection (FBP) based method with a Hann filter of 4 mm full-width at half-maximum.

The intensity of FMISO accumulation was assessed by a semi-quantitative procedure. The standardized uptake values (SUVs) were calculated as [tissue radioactivity (Bq/mL)] × [body weight (g)] / [injected radioactivity (Bq)]. In this study, the SUV of the cerebellar cortex was adopted as a normal reference, as previously demonstrated [30]. Circular regions of interests (ROIs) 10 mm in diameter were created on both sides of the cerebellar cortex (5 ROIs per hemisphere) under MRI reference. The average values within the ROIs were used as the normal reference SUV. A circular ROI 10 mm in diameter for lesions was manually located to enclose the area of the highest uptake in the lesion to obtain the maximum SUV (SUVmax). Then, Tumor-to-Normal (T/N) ratio was calculated by dividing the SUVmax of the tumor by the mean SUV of the normal cerebellar cortex.

In addition, FMISO accumulation volume was measured using a software package (Metavol) that we developed previously to conduct volume-based analyses of PET images [31]. The FMISO accumulation volume was automatically contoured using a threshold of 1.3-fold cerebellar mean SUV [19]. If the automatically-generated volume contained normal brain area, the non-tumoral area was carefully removed by the operator, who referred to the co-registered MRI using a function implemented in Metavol. An experienced nuclear medicine physician confirmed the final FMISO accumulation volume, defined as hypoxic volume.

FMISO response by BEV administration was assessed by both visual inspection and semi-quantitative values. In visual inspection, two nuclear medicine physicians (T.T. and K.H.), who were blinded to the clinical situation of patients treated by BEV, interpreted the FMISO PET response. Their evaluations were classified into three categories according to FMISO accumulation volume before BEV administration and after 3–4 courses BEV: decreased, stable, and increased. Simultaneously, changes of the FMISO T/N ratio and hypoxic volume were calculated as semi-quantitative values. Patients were classified as FMISO responders if FMISO accumulation was visually decreased, and the FMISO T/N ratio decreased with a more than 25% decrease in the hypoxic volume after the administration of BEV.

### Statistical analysis

R statistical software, version 3.0.3, was used to conduct all statistical analyses and create graphical images. Continuous variable data were expressed as median values, and the Mann-Whitney U-test was used to compare the median values of the two groups. To determine the correlation between two volumetric values, rho(*r*) and *P*-values were calculated using Spearman’s rank correlation coefficient; the scatterplot was presented with least-squares linear regression. Estimated survival curves were shown by the Kaplan-Meier method, and log-rank tests were used for comparison. To analyze the association of predictive pre-treatment factors of BEV treatment with overall survival, univariate analysis and multivariate analysis were carried out using the Cox proportional hazard model. The analyzed characteristics included age at recurrence, primary diagnosis (GBM or non-GBM), Karnofsky performance status (KPS) at BEV administration (30–60% or more than 70%), duration between onset to recurrence, recurrence pattern (local recurrence or distal recurrence), combination with TMZ, contrasted-enhancement volume at MRI, FMISO T/N ratio, and FMISO hypoxic volume. The proportional hazard assumption was tested for each model. In multivariate analysis, the factors for which the P-value was below 0.1 in univariate analysis were selected.

*P*-values less than 0.05 were considered statistically significant.

## Results

### ^18^F-FMISO PET appearances of recurrent gliomas

All recurrence regions before the administration of BEV, which were defined at baseline examination, were clearly detected as FMISO accumulating lesions regardless of histological malignancy on primary diagnosis, as shown in Table 1. The median SUVmax and T/N ratio of FMISO of recurrent tumors were 3.6 (range, 2.4–6.4) and 2.8 (range, 1.9–4.3), respectively. No statistically significant differences in SUVmax and T/N ratio were observed with regard to initial histological diagnosis. The median SUVmax and T/N ratio of the recurrent tumor were 3.4 and 2.5, respectively, in the patients with initial GBM, whereas that of the recurrent tumor were 4.3 and 3.1, respectively, in the patients with initial non-GBM.

**Table 1:** Patient Characteristics, imaging findings, response evaluation, and prognosis.

Pt No.	Age/Sex	Initial Dx	FMISO baseline		FMISO after BEV		MRI(Gd) tumor Vol (mL)		MRI response^a^	FMISO response	OS (months)
			T/N	Vol (mL)	T/N	Vol (mL)	baseline	After BEV			
1	65/M	GBM	4.0	35.5	4.3	48.3	37.5	20.5	PR	non-responder	3.1
2	67/F	DA	3.7	33.5	4.8	38.5	45.0	16.6	PR	non-responder	6.3
3	68/M	GBM	4.3	15.6	5.0	38.9	18.3	18.7	SD	non-responder	4.8
4	62/M	GBM	3.3	25.4	2.3	12.4	13.6	1.2	PR	responder	12.4
5	66/F	AA	2.9	17.2	2.1	8.8	24.2	3.0	PR	responder	9.0
6	68/F	AA	3.5	37.0	3.9	49.7	58.2	12.7	PR	non-responder	5.7
7	38/F	GBM	1.9	8.9	3.8	62.6	12.4	63.1	PD	non-responder	6.4
8	68/F	AA	1.9	2.2	2.9	5.7	3.2	3.5	SD	non-responder	9.5
9	61/M	AA	3.3	12.3	3.6	29.1	23.3	21.4	SD	non-responder	4.0
10	62/F	GBM	2.0	6.9	1.2	0	11.9	2.5	PR	responder	17.0*
11	58/F	AA	2.3	24.0	1.8	8.1	40.1	20.3	PR	responder	8.1*
12	71/M	GBM	1.9	0.8	2.0	2.7	1.1	0.5	PR	non-responder	8.4*
13	47/M	GBM	2.3	16.0	1.7	2.2	31.4	7.9	PR	responder	10.4*
14	56/F	GBM	2.2	6.0	1.7	1.5	3.7	0.8	PR	responder	5.6
15	27/F	GBM	2.2	9.6	1.7	2.2	22.8	9.7	PR	responder	9.3*
16	70/M	GBM	3.4	44.4	2.5	38.8	56.6	6.1	PR	non-responder	5.5
17	76/F	GBM	2.8	16.9	2.1	9.8	14.5	2.9	PR	responder	2.9*
18	43/M	GBM	2.9	38.3	1.9	7.5	41.5	14.8	PR	responder	3.3*

AA, anaplastic astrocytoma; BEV, Bevacizumab; DA, diffuse astrocytoma; Dx, diagnosis; GBM, glioblastoma multiforme; Gd, gadolinium; OS. overall survival; Pt, patient; RPA, recursive partitioning analysis; Vol, volume.

^a^MRI responses were assessed by RANO criteria [10].

*Censoring at time of reporting

**Fig 1:**
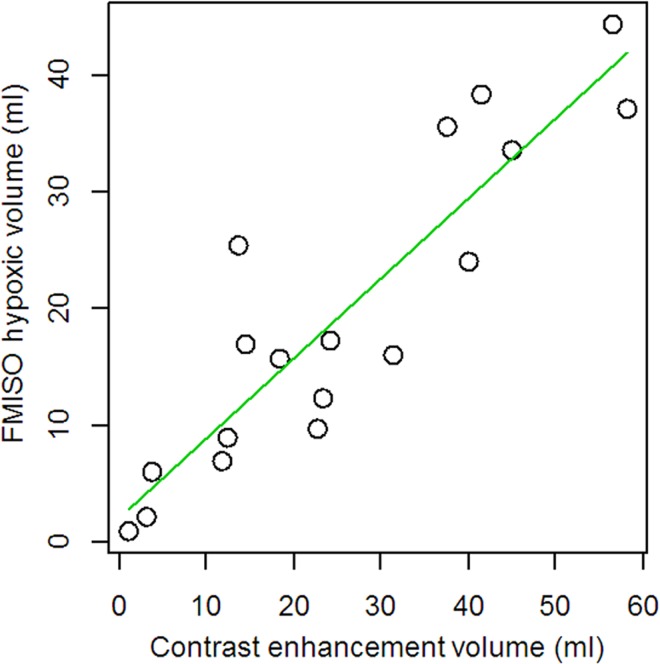
Correlation between contrast-enhanced tumor volumes and FMISO hypoxic volumes before BEV treatment. There are strong positive correlation between these two volumetric values (*r* = 0.89, *P* < 0.001).

The median FMISO hypoxic volume was 16.4 mL (range: 0.8–38.3 mL). In every case, FMISO accumulating regions closely resembled contrast enhancement regions on MRI. The tumor volume, which was calculated by contrast enhancement, strongly correlated with the hypoxic volume (*r* = 0.89, *p* < 0.001, Fig 1A).

### Therapeutic effects of BEV assessed by MRI and FMISO PET

**Fig 2:**
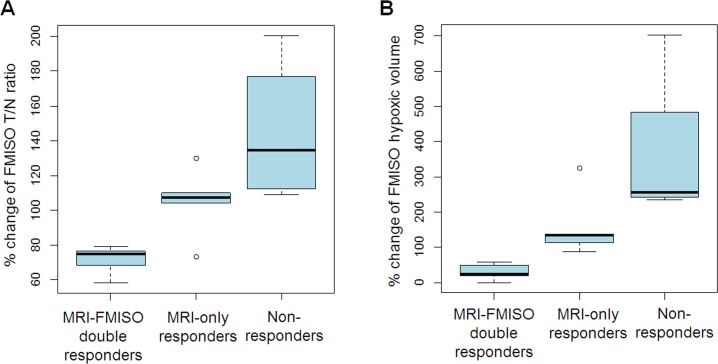
FMISO Accumulation change after 3–4 courses BEV treatment. Percentage change of FMISO accumulation T/N ratio (A) and volume (B) according to the classification by MRI and FMISO PET responses.

Based on MRI RANO criteria, 14 out of 18 (78%) patients demonstrated partial response after 3–4 courses of BEV treatment, while 4 patients (22%) did not show any therapeutic response (Table 1). In these 4 patients, defined as “non-responders”, FMISO T/N ratio and hypoxic volume also increased. Percentage change of the T/N ratio ranged from 109% to 201% (median 134%) and that of hypoxic volume ranged from 236% to 703% (median 255%). On the other hand, 14 “MRI-responder” patients had variable FMISO accumulation responses. Of these 14 patients, 9 were also FMISO responders. We named them as “MRI-FMISO double responders”. The other 5 patients were FMISO non-responders and we named them as “MRI-only responders”. Percentage change of the T/N ratio and hypoxic volume of MRI-FMISO double responders ranged from 58.4% to 79.1% (median 75.1%) and from 0% to 57.8% (median 24.5%), respectively, whereas percentage change of the T/N ratio and hypoxic volume of MRI-only responders ranged from 73.1% to 130% (median 107%) and from 87.4% to 325%, respectively (Fig 2). Ultimately, according to changes on MRI and FMISO PET appearance, we divided the patients into three groups: MRI-FMISO double responders (n = 9), MRI-only responders (n = 5), and non-responders (n = 4).

**Fig 3:**
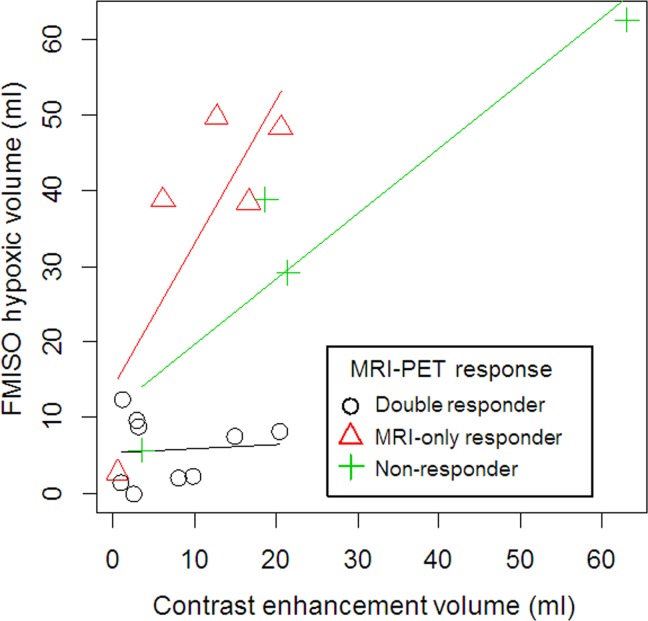
Correlation between contrast-enhanced tumor volumes and FMISO hypoxic volumes 3 to 4 courses after BEV treatment according to treatment response groups. Circles, Triangles, and Crosses represent MRI-FMISO double responders, MRI-only responders, and non-responders, respectively.

The correlation between FMISO hypoxic volume and contrast-enhanced tumor volume based on gadolinium T1-weighted MRI was quite intriguing. The scatterplot is shown in Fig 3. Although the significant correlation between these two volumetric values (*r* = 0.62, *P* = 0.007) was still observed, as with before BEV treatment, we found some trends according to therapeutic response groups. Notably, in the MRI-only responder group, hypoxic volumes were relatively higher than contrast-enhanced tumor volumes (triangle in Fig 3).

**Fig 4:**
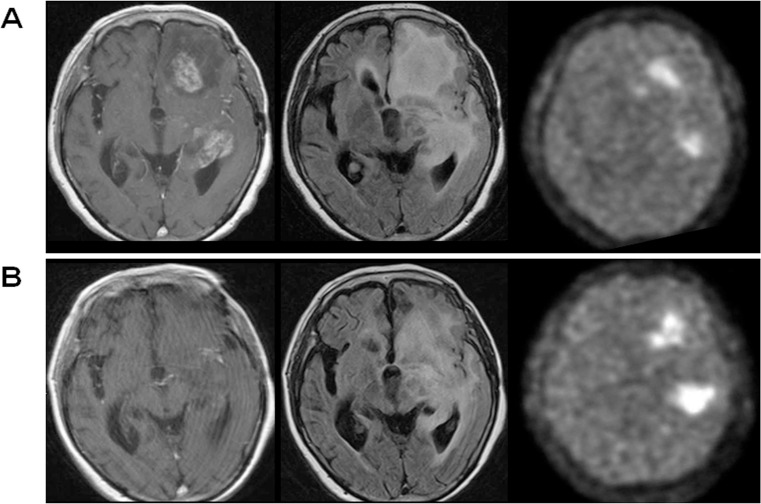
Representative cases of MRI-only responder (Case 2). Gadolinium-enhanced T1-weigted images (left panels), FLAIR images (middle panels), and FMISO PET images (right panels) are shown, and upper panels show the case before BEV treatment and lower panels show the case three courses after BEV administration. After BEV treatment, contrast-enhanced lesion and FLAIR hyperintensity lesion were significantly decreased, whereas FMISO accumulation was increased in both T/N ratio (from 3.70 to 4.81) and volume (from 33.5 mL to 38.5 mL).

**Fig 5:**
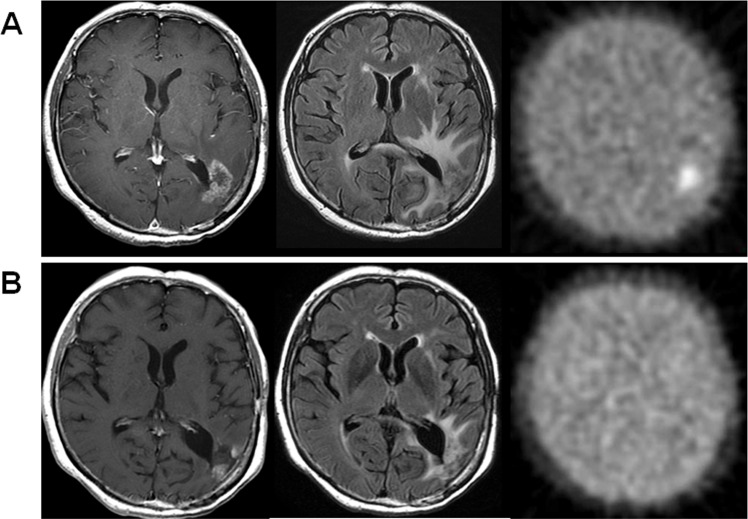
Representative cases of MRI-FMISO double responder (Case 10). The panel composition is same as Fig 4. After BEV treatment, contrast-enhanced lesion and FLAIR hyperintensity lesions were decreased. Simultaneously, FMISO accumulation was disappeared.

Additionally, we investigated whether pre-treatment tumor volume had an impact on BEV’s therapeutic effect. We observed no significant differences in contrast-enhanced pre-treatment tumor volume between responders and non-responders on MRI evaluation and FMISO evaluation. The median tumor volume of MRI responders was 27.8 mL and that of MRI non-responders was 15.4 mL. The median tumor volume of FMISO responders was 16.0 mL and that of FMISO non-responders was 20.8 mL. There was no volumetric difference among the two groups at baseline (*P* = 0.19 in MRI assessment, *P* = 0.55 in FMISO assessment), indicating that the tumor size prior to treatment did not affect the treatment response by BEV. Representative images are shown (Figs 4 and 5).

### Prognostic prediction by FMISO PET

**Fig 6:**
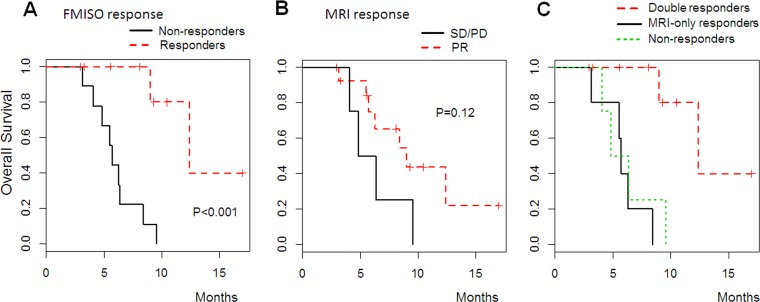
Overall survival according to treatment response by MRI and FMISO PET. Kaplan-Meier curves separated by FMISO response (A). Censored observation are marked with vertical bars. In addition, OS curves were shown according to MRI response (B) and three groups classified by MRI and FMISO response (C); MRI-FMISO double responders (red broken line), MRI-only responders (black solid line), and non-responders (green dot line).

FMISO responders had significantly longer OS than that of FMISO non-responders (*P* <0.001) (Fig 6A). The median OS of FMISO responders was 12.4 months, whereas that of FMISO non-responders was 5.7 months. On the other hand, when BEV response was classified based on MRI evaluations, we did not observe a significant difference in OS between responders and non-responders (*P* = 0.12) (Fig 6B). In addition, of the three groups (MRI-FMISO double responders, MRI-only responders, and non-responders), the prognosis of MRI-FMISO double responders was much better than that of the other two groups. Moreover, the OS of MRI-only responders (median OS, 5.7 months) closely resembled that of non-responders (median OS, 4.8 months) (*P* = 0.58, Fig 6C).

We further investigated a pre-treatment parameter which is predictive of overall survival by Cox proportional hazard models (Table 2). Among each factor, the duration from onset to recurrence and FMISO T/N ratio before BEV were significant predictors in univariate analysis. In addition, multivariate analysis showed that poor KPS at BEV treatment and high FMISO T/N ratios were significant poor prognostic factors. Interestingly, contrast enhanced-tumor volume and FMISO hypoxic volume were not correlated with patient’s prognosis with BEV treatment.

**Table 2:** Potential pre-BEV treatment predictors of overall survival in the patient recurrent high-grade glioma by BEV in Cox hazard model.

Factors	Univariate analysis			Multivariate analysis		
	Hazard ratio	95% CI	P-value	Hazard ratio	95% CI	P-value
Age [year]*	1.062	0.978–1.153	0.155	-	-	-
**Primary Diagnosis [non-GBM]**	1.832	0.525–6.389	0.342	-	-	-
**KPS at BEV [70–100%]**	0.272	0.074–1.005	0.051	0.11	0.017–0.724	**0.022**
Duration between onset to recurrence [months]*	1.075	1.005–1.151	**0.036**	0.969	0.874–1.074	0.55
**Recurrence pattern [local]**	0.682	0.196–2.382	0.55	-	-	-
**Combined with TMZ [yes]**	0.189	0.034–1.041	0.056	1.083	0.097–12.12	0.95
Gd volume before BEV [mL]*	1.03	0.989–1.072	0.152	-	-	-
FMISO T/N ratio before BEV*	3.667	1.412–9.521	**0.008**	13.86	1.75–109.8	**0.013**
FMISO volume before BEV [mL]*	1.049	0.994–1.106	0.08	0.924	0.832–1.028	0.15

BEV, Bevacizumab; CI, confidence interval; FMISO, 18F-fluoromisonidazole; GBM, Glioblastoma multiforme; Gd, Gadorinium; KPS, Karnofsky performance status; TMZ, Temozolomide; T/N, Tumor-to Normal.

*continuous variable

## Discussions

In this study, we found that FMISO PET imaging can be used for early detection of true responders to BEV treatment. Previously, ^18^F-FET [15,16] and ^18^F-FDOPA [17] were reported to be promising prognostic metabolic biomarkers in predicting the response of patients with recurrent high-grade glioma to BEV treatment. These amino acid tracers are not dependent upon blood-brain barrier (BBB) disruption and they can detect the extent of the active tumor [32,33]. Since the normalization of tumor vasculature can be induced by BEV treatment [25], these tracers may theoretically delineate the tumor infiltrative area after BEV treatment regardless of contrast enhancement of the tumor. These previous studies demonstrated that tumor metabolic volume changes could be strong predictors of prognosis for patients who received BEV treatment. However, both ^18^F-FET and ^18^F-FDOPA failed to show a correlation between tracer uptake (SUV) changes and prognosis [15–17]. In addition, although volumetric changes in these studies were significant between metabolic responders and non-responders, tracer uptake volumes also decreased in non-responders, and appropriate cut-off values had to be determined for each study.

Compared to these amino acid tracers, the FMISO PET changes were more comprehensible. By showing decreased FMISO accumulation tumor values and volumes in FMISO responders, FMISO PET could predict a good response to treatment with BEV. On the other hand, tumors from FMISO non-responders showed increased FMISO hypoxic volumes and poorer prognoses. Therefore, by analyzing changes in FMISO accumulation, clinicians could easily determine whether BEV is likely to be effective for each tumor or not. Our results strongly suggest that, for the treatment of glioma with BEV, FMISO PET may be a superior predictive examination tool, compared to other tracers.

In this study, we also investigated the correlation between FMISO hypoxic volume and contrast-enhanced tumor volume based on MRI. As expected, before BEV treatment, the hypoxic volume was almost equal to the contrast-enhanced tumor volume (Fig 1A; r = 0.89). On the other hand, we observed some interesting discrepancies after BEV treatment between hypoxic volume and contrast-enhanced tumor volume (Fig 1B). In particular, in the MRI-only responder group, hypoxic volumes were higher than contrast-enhanced volumes. Importantly, MR responders in this series experienced not only decreased contrast-enhancement but also decreased or stable T2/FLAIR high-intensity lesions after 3–4 courses of BEV. We observed no survival difference between non-responders and MRI-only responders. Therefore, after BEV treatment, clinicians can more precisely evaluate tumor activity by analyzing FMISO accumulation compared with the analysis of the appearance of tumors on MRI. Furthermore, it is important that, after BEV administration, the evaluation of this volumetric discrepancy between contrast-enhanced MRI and FMISO PET may also be predictive of prognosis when a baseline examination is not available.

Bevacizumab inhibits VEGF and can induce normalization of tumor vasculature and decrease blood vessel diameter and permeability, leading to increased perfusion and oxygenation [25,34]. This can occur because VEGF is a principal mediator of angiogenesis and serves to recruit and induce proliferation of vascular endothelial cells [35]. Therefore, by measuring the hypoxic conditions, one should be able to reliably evaluate the effects of BEV treatment [26,36]. FMISO accumulates in hypoxic, yet still viable, cells [37]. FMISO accumulation occurs when the partial O_2_ is less than 10–20 mmHg [20,38] and substantial retention of FMISO occurs when it is less than 3 mmHg [39]. Since the intensity of FMISO accumulation depends on the degree of tissue hypoxia [39], decreasing FMISO accumulation should indicate the efficacy of BEV for the treatment of recurrent glioma. In addition, single-arm phase II trials demonstrated that increase in tumor blood perfusion in patients with GBM treated with cediranib, which is another anti-VEFF agents, was associated with the improvement prognosis [40,41]. These studies showed that the positive correlation between the increase tumor blood perfusion and improvement tumor oxygenation. The GBM patients whose tumor blood perfusion and oxygenation increased during treatment with cediranib survived longer than those without such an increase. Unfortunately, we did not investigate the tumor blood perfusion in the current study. We consider that future studies should need the evaluation of the association between tumor blood perfusion and hypoxic condition detected by FMSIO PET in the patients with GBM treated by BEV, for the proper selection of the patients who are likely to optimally benefit from BEV therapy.

Our study was able to identify gliomas with retention of hypoxia after BEV treatment regardless of MRI appearance. These “FMISO non-responders” would become refractory to BEV treatment in short term. In a recent study, Hu *et al* showed that BEV-resistant tumors exhibited increased hypoxic markers, such as hypoxic inducible factor 1α (HIF-1α), carbonic anhydrase 9 (CA9), and their downstream target gene BNIP3, compared with before BEV treatment. They advocated that increased tumor hypoxia during anti-angiogenic therapy induces tumor cell autophagy as a cytoprotective adaptive response, thereby promoting treatment resistance [27]. The result of their basic investigation strongly supports our clinical results. Since FMISO can detect intratumoral hypoxic conditions regardless of tumor vascularity, our results suggest that, compared to other amino acid tracers, FMISO accumulation is a more sensitive detector for BEV-resistant tumors.

In addition, among pre-treatment parameters, the FMISO T/N ratio is a strong significant predictor of overall survival after BEV treatment by multivariate analysis. As described above, the intensity of FMISO accumulation is proportional to the level of hypoxia [39]. Although hypoxic tumor cells are known to affect resistance to chemotherapy [42], there has been no previous investigation into whether the extent of hypoxia would influence the effect of anti-angiogenic treatments, such as BEV. According to our result, the outcome of BEV treatment is not related to hypoxic volume but hypoxic intensity, indicating that the extent of hypoxia might be a potentially important factor for drug resistance to BEV in recurrent malignant gliomas.

## Conclusions

Despite the small study population and the study’s retrospective nature, we demonstrated that, by analyzing changes in the appearance of tumors, FMISO PET can be a strong and useful predictive tool for assessing response to BEV treatment in patients with recurrent high-grade gliomas. Increasing FMISO accumulation intensities and volumes after 3–4 courses of BEV might indicate a BEV-resistant tumor. Subsequent validation studies and clinical trials are needed to further evaluate the diagnostic potential of FMISO PET in the treatment of glioma with BEV.

## References

[1] Chinot OL, Wick W, Mason W, Henriksson R, Saran F, Nishikawa R, et al Bevacizumab plus radiotherapy-temozolomide for newly diagnosed glioblastoma. N Engl J Med. 2014;370(8): 709–722. 10.1056/NEJMoa1308345 24552318

[2] Gilbert MR, Dignam JJ, Armstrong TS, Wefel JS, Blumenthal DT, Vogelbaum MA, et al A randomized trial of bevacizumab for newly diagnosed glioblastoma. N Engl J Med. 2014;370(8): 699–708. 10.1056/NEJMoa1308573 24552317 PMC4201043

[3] Friedman HS, Prados MD, Wen PY, Mikkelsen T, Schiff D, Abrey LE, et al Bevacizumab alone and in combination with irinotecan in recurrent glioblastoma. J Clin Oncol. 2009;27(28): 4733–4740. 10.1200/JCO.2008.19.8721 19720927

[4] Kreisl TN, Kim L, Moore K, Duic P, Royce C, Stroud I, et al Phase II trial of single-agent bevacizumab followed by bevacizumab plus irinotecan at tumor progression in recurrent glioblastoma. J Clin Oncol. 2009;27(5): 740–745. 10.1200/JCO.2008.16.3055 19114704 PMC2645088

[5] Macdonald DR, Cascino TL, Schold SC Jr., Cairncross JG. Response criteria for phase II studies of supratentorial malignant glioma. J Clin Oncol. 1990;8(7): 1277–1280. 10.1200/jco.1990.8.7.1277 2358840

[6] Norden AD, Young GS, Setayesh K, Muzikansky A, Klufas R, Ross GL, et al Bevacizumab for recurrent malignant gliomas: efficacy, toxicity, and patterns of recurrence. Neurology. 2008;70(10): 779–787. 10.1212/01.wnl.0000304121.57857.38 18316689

[7] Schaub C, Greschus S, Seifert M, Waha A, Blasius E, Rasch K, et al FLAIR-only progression in bevacizumab-treated relapsing glioblastoma does not predict short survival. Oncology. 2013;85(3): 191–195. 10.1159/000354692 24008924

[8] Wick A, Dorner N, Schafer N, Hofer S, Heiland S, Schemmer D, et al Bevacizumab does not increase the risk of remote relapse in malignant glioma. Ann Neurol. 2011;69(3): 586–592. 10.1002/ana.22336 21446027

[9] Nowosielski M, Wiestler B, Goebel G, Hutterer M, Schlemmer HP, Stockhammer G, et al Progression types after antiangiogenic therapy are related to outcome in recurrent glioblastoma. Neurology. 2014;82(19): 1684–1692. 10.1212/WNL.0000000000000402 24727314

[10] Wen PY, Macdonald DR, Reardon DA, Cloughesy TF, Sorensen AG, Galanis E, et al Updated response assessment criteria for high-grade gliomas: response assessment in neuro-oncology working group. J Clin Oncol. 2010;28(11): 1963–1972. 10.1200/JCO.2009.26.3541 20231676

[11] Boxerman JL, Zhang Z, Safriel Y, Larvie M, Snyder BS, Jain R, et al Early post-bevacizumab progression on contrast-enhanced MRI as a prognostic marker for overall survival in recurrent glioblastoma: results from the ACRIN 6677/RTOG 0625 Central Reader Study. Neuro Oncol. 2013;15(7): 945–954. 10.1093/neuonc/not049 23788270 PMC3688018

[12] Ellingson BM, Cloughesy TF, Lai A, Nghiemphu PL, Mischel PS, Pope WB. Quantitative volumetric analysis of conventional MRI response in recurrent glioblastoma treated with bevacizumab. Neuro Oncol. 2011;13(4): 401–409. 10.1093/neuonc/noq206 21324937 PMC3064698

[13] Huang RY, Rahman R, Hamdan A, Kane C, Chen C, Norden AD, et al Recurrent glioblastoma: volumetric assessment and stratification of patient survival with early posttreatment magnetic resonance imaging in patients treated with bevacizumab. Cancer. 2013;119(19): 3479–3488. 10.1002/cncr.28210 23821555

[14] Hutterer M, Hattingen E, Palm C, Proescholdt MA, Hau P. Current standards and new concepts in MRI and PET response assessment of antiangiogenic therapies in high-grade glioma patients. Neuro Oncol. 2015;17(6): 784–800. 10.1093/neuonc/nou322 25543124 PMC4483117

[15] Galldiks N, Rapp M, Stoffels G, Fink GR, Shah NJ, Coenen HH, et al Response assessment of bevacizumab in patients with recurrent malignant glioma using [18F]Fluoroethyl-L-tyrosine PET in comparison to MRI. Eur J Nucl Med Mol Imaging. 2013;40(1): 22–33. 10.1007/s00259-012-2251-4 23053325

[16] Hutterer M, Nowosielski M, Putzer D, Waitz D, Tinkhauser G, Kostron H, et al O-(2-18F-fluoroethyl)-L-tyrosine PET predicts failure of antiangiogenic treatment in patients with recurrent high-grade glioma. J Nucl Med. 2011;52(6): 856–864. 10.2967/jnumed.110.086645 21622893

[17] Schwarzenberg J, Czernin J, Cloughesy TF, Ellingson BM, Pope WB, Grogan T, et al Treatment response evaluation using 18F-FDOPA PET in patients with recurrent malignant glioma on bevacizumab therapy. Clin Cancer Res. 2014;20(13): 3550–3559. 10.1158/1078-0432.CCR-13-1440 24687922 PMC4079729

[18] Koch CJ, Evans SM. Non-invasive PET and SPECT imaging of tissue hypoxia using isotopically labeled 2-nitroimidazoles. Adv Exp Med Biol. 2003;510(285–292. 12580442 10.1007/978-1-4615-0205-0_47

[19] Hirata K, Terasaka S, Shiga T, Hattori N, Magota K, Kobayashi H, et al (1)(8)F-Fluoromisonidazole positron emission tomography may differentiate glioblastoma multiforme from less malignant gliomas. Eur J Nucl Med Mol Imaging. 2012;39(5): 760–770. 10.1007/s00259-011-2037-0 22307533

[20] Swanson KR, Chakraborty G, Wang CH, Rockne R, Harpold HL, Muzi M, et al Complementary but distinct roles for MRI and 18F-fluoromisonidazole PET in the assessment of human glioblastomas. J Nucl Med. 2009;50(1): 36–44. 10.2967/jnumed.108.055467 19091885 PMC4154624

[21] Kawai N, Maeda Y, Kudomi N, Miyake K, Okada M, Yamamoto Y, et al Correlation of biological aggressiveness assessed by 11C-methionine PET and hypoxic burden assessed by 18F-fluoromisonidazole PET in newly diagnosed glioblastoma. Eur J Nucl Med Mol Imaging. 2011;38(3): 441–450. 10.1007/s00259-010-1645-4 21072512

[22] Spence AM, Muzi M, Swanson KR, O'Sullivan F, Rockhill JK, Rajendran JG, et al Regional hypoxia in glioblastoma multiforme quantified with [18F]fluoromisonidazole positron emission tomography before radiotherapy: correlation with time to progression and survival. Clin Cancer Res. 2008;14(9): 2623–2630. 10.1158/1078-0432.CCR-07-4995 18451225 PMC4415875

[23] Cher LM, Murone C, Lawrentschuk N, Ramdave S, Papenfuss A, Hannah A, et al Correlation of hypoxic cell fraction and angiogenesis with glucose metabolic rate in gliomas using 18F-fluoromisonidazole, 18F-FDG PET, and immunohistochemical studies. J Nucl Med. 2006;47(3): 410–418. 16513609

[24] Barajas RF Jr., Pampaloni MH, Clarke JL, Seo Y, Savic D, Hawkins RA, et al Assessing Biological Response to Bevacizumab Using 18F-Fluoromisonidazole PET/MR Imaging in a Patient with Recurrent Anaplastic Astrocytoma. Case Rep Radiol. 2015;2015(731361 10.1155/2015/731361 25793136 PMC4352456

[25] Weathers SP, de Groot J. Resistance to antiangiogenic therapy. Curr Neurol Neurosci Rep. 2014;14(5): 443 10.1007/s11910-014-0443-y 24652451

[26] Valable S, Petit E, Roussel S, Marteau L, Toutain J, Divoux D. et al Complementary information from magnetic resonance imaging and (18)F-fluoromisonidazole positron emission tomography in the assessment of the response to an antiangiogenic treatment in a rat brain tumor model. Nucl Med Biol. 2011;38(6): 781–793. 10.1016/j.nucmedbio.2011.01.010 21843775

[27] Hu YL, DeLay M, Jahangiri A, Molinaro AM, Rose SD, Carbonell WS, et al Hypoxia-induced autophagy promotes tumor cell survival and adaptation to antiangiogenic treatment in glioblastoma. Cancer Res. 2012;72(7): 1773–1783. 10.1158/0008-5472.CAN-11-3831 22447568 PMC3319869

[28] Narita T, Aoyama H, Hirata K, Onodera S, Shiga T, Kobayashi H, et al Reoxygenation of glioblastoma multiforme treated with fractionated radiotherapy concomitant with temozolomide: changes defined by 18F-fluoromisonidazole positron emission tomography: two case reports. Jpn J Clin Oncol. 2012;42(2): 120–123. 10.1093/jjco/hyr181 22198964

[29] Stupp R, Mason WP, van den Bent MJ, Weller M, Fisher B, Taphoorn MJ, et al Radiotherapy plus concomitant and adjuvant temozolomide for glioblastoma. N Engl J Med. 2005;352(10): 987–996. 10.1056/NEJMoa043330 15758009

[30] Bruehlmeier M, Roelcke U, Schubiger PA, Ametamey SM. Assessment of hypoxia and perfusion in human brain tumors using PET with F-18-fluoromisonidazole and O-15-H2O. Journal of Nuclear Medicine. 2004;45(11): 1851–1859. 15534054

[31] Hirata K, Kobayashi K, Wong KP, Manabe O, Surmak A, Tamaki N, et al A semi-automated technique determining the liver standardized uptake value reference for tumor delineation in FDG PET-CT. PLoS One. 2014;9(8): e105682 10.1371/journal.pone.0105682 25162396 PMC4146536

[32] Pauleit D, Floeth F, Hamacher K, Riemenschneider MJ, Reifenberger G, Muller HW, et al O-(2-[18F]fluoroethyl)-L-tyrosine PET combined with MRI improves the diagnostic assessment of cerebral gliomas. Brain. 2005;128(Pt 3): 678–687. 10.1093/brain/awh399 15689365

[33] Fueger BJ, Czernin J, Cloughesy T, Silverman DH, Geist CL, Walter MA, et al Correlation of 6-18F-fluoro-L-dopa PET uptake with proliferation and tumor grade in newly diagnosed and recurrent gliomas. J Nucl Med. 2010;51(10): 1532–1538. 10.2967/jnumed.110.078592 20847166

[34] Thomas AA, Omuro A. Current role of anti-angiogenic strategies for glioblastoma. Curr Treat Options Oncol. 2014;15(4): 551–566. 10.1007/s11864-014-0308-2 25173555

[35] Kargiotis O, Rao JS, Kyritsis AP. Mechanisms of angiogenesis in gliomas. J Neurooncol. 2006;78(3): 281–293. 10.1007/s11060-005-9097-6 16554966

[36] Bell C, Dowson N, Fay M, Thomas P, Puttick S, Gal Y, et al Hypoxia imaging in gliomas with 18F-fluoromisonidazole PET: toward clinical translation. Semin Nucl Med. 2015;45(2): 136–150. 10.1053/j.semnuclmed.2014.10.001 25704386

[37] Koh WJ, Rasey JS, Evans ML, Grierson JR, Lewellen TK, Graham MM, et al Imaging of hypoxia in human tumors with [F-18]fluoromisonidazole. Int J Radiat Oncol Biol Phys. 1992;22(1): 199–212. 1727119 10.1016/0360-3016(92)91001-4

[38] Piert M, Machulla H, Becker G, Stahlschmidt A, Patt M, Aldinger P, et al Introducing fluorine-18 fluoromisonidazole positron emission tomography for the localisation and quantification of pig liver hypoxia. Eur J Nucl Med. 1999;26(2): 95–109. 9933343 10.1007/s002590050365

[39] Rasey JS, Nelson NJ, Chin L, Evans ML, Grunbaum Z. Characteristics of the binding of labeled fluoromisonidazole in cells in vitro. Radiat Res. 1990;122(3): 301–308. 2356284

[40] Batchelor TT, Gerstner ER, Emblem KE, Duda DG, Kalpathy-Cramer J, Snuderl M, et al Improved tumor oxygenation and survival in glioblastoma patients who show increased blood perfusion after cediranib and chemoradiation. Proc Natl Acad Sci U S A. 2013;110(47): 19059–19064. 10.1073/pnas.1318022110 24190997 PMC3839699

[41] Sorensen AG, Emblem KE, Polaskova P, Jennings D, Kim H, Ancukiewicz M, et al Increased survival of glioblastoma patients who respond to antiangiogenic therapy with elevated blood perfusion. Cancer Res. 2012;72(2): 402–407. 10.1158/0008-5472.CAN-11-2464 22127927 PMC3261301

[42] Liang BC. Effects of hypoxia on drug resistance phenotype and genotype in human glioma cell lines. J Neurooncol. 1996;29(2): 149–155. 8858520 10.1007/BF00182138

